# Inheritance of HLA-Cw7 Associated With Autism Spectrum Disorder (ASD)

**DOI:** 10.3389/fpsyt.2019.00612

**Published:** 2019-09-11

**Authors:** Terry Harville, Bobbie Rhodes-Clark, Sirish C. Bennuri, Leanna Delhey, John Slattery, Marie Tippett, Rebecca Wynne, Shannon Rose, Stephen Kahler, Richard E. Frye

**Affiliations:** ^1^Department of Pathology, University of Arkansas for Medical Sciences, Little Rock, AR, United States; ^2^Department of Internal Medicine, University of Arkansas for Medical Sciences, Little Rock, AR, United States; ^3^Department of Pediatrics, College of Medicine, University of Arkansas for Medical Sciences, Little Rock, AR, United States; ^4^School of Public Health, University of Arkansas for Medical Sciences, Little Rock, AR, United States; ^5^Arkansas Children’s Research Institute, Little Rock, AR, United States; ^6^BioRosa Technologies Inc, San Francisco, CA, United States; ^7^National Center for Toxicological Research, Jefferson, AR, United States; ^8^Barrow Neurological Institute at Phoenix Children’s Hospital, Phoenix, AZ, United States; ^9^Department of Child Health, University of Arizona College of Medicine, Phoenix, AZ, United States

**Keywords:** autism (ASD), HLA, immune system, natural killer cells, innate immnuity

## Abstract

Autism spectrum disorder (ASD) is a behaviorally defined disorder that is now thought to affect approximately 1 in 69 children in the United States. In most cases, the etiology is unknown, but several studies point to the interaction of genetic predisposition with environmental factors. The immune system is thought to have a causative role in ASD, and specific studies have implicated T lymphocytes, monocytes, natural killer (NK) cells, and certain cytokines. The human leukocyte antigen (HLA) system is involved in the underlying process for shaping an individual’s immune system, and specific HLA alleles are associated with specific diseases as risk factors. In this study, we determine whether a specific HLA allele was associated with ASD in a large cohort of patients with ASD. Identifying such an association could help in the identification of immune system components which may have a causative role in specific cohorts of patients with ASD who share similar specific clinical features. Specimens from 143 patients with ASD were analyzed with respect to race and ethnicity. Overall, HLA-Cw7 was present in a much greater frequency than expected in individuals with ASD as compared to the general population. Further, the cohort of patients who express HLA-Cw7 shares specific immune system/inflammatory clinical features including being more likely to have allergies, food intolerances, and chronic sinusitis as compared to those with ASD who did not express HLA-Cw7. HLA-Cw7 has a role in stimulating NK cells. Thus, this finding may indicate that chronic over-activation of NK cells may have a role in the manifestation of ASD in a cohort of patients with increased immune system/inflammatory features.

## Introduction

Autism spectrum disorder (ASD) is a behaviorally defined disorder that affects approximately 1 in 69 children in the United States (1). The definition of ASD is outlined in the Diagnostic and Statistical Manual of Mental Disorders (DSM)-5 which requires that those diagnosed exhibit deficits in social communication along with the presence of restricted interests and/or repetitive behaviors and/or sensory disorders. In addition, the symptoms must significantly interfere with the individual’s ability to function properly in school, work, and other areas of life (2). Despite decades of research, the etiology(ies) of ASD is(are) still unknown (3), but epidemiological studies suggest that genetic predisposition interacts with environmental influences to trigger the biological events that results in ASD (4). Several physiological systems that are known to be influenced by environmental factors have been implicated in ASD, including redox and mitochondrial metabolism, as well as, immune dysregulation (3, 4).

ASD is described as a “spectrum” due to the variability in the extent and severity of behavioral and medical symptoms. The diagnostic criteria for ASD is wide enough to capture an overall phenotype, but the variability in symptoms and severity, as well as, specific laboratory results indicates that multiple etiologies may be responsible, including one that involves immune dysfunction (5). One of the challenges is to define potential subtypes using behavioral and physiological biomarkers in order to better define the disorder and identify treatment targets.

Many studies have indicated involvement of the immune system in ASD. Early studies identified family histories of autoimmune disease, specific HLA associations, and autoantibodies to a wide range of brain and non-brain tissues in children with ASD (6–13). Other studies have demonstrated a reduction in immunoglobulin production (14) and a positive therapeutic response to intravenous immunoglobulin treatment (15). Perhaps the most pivotal study from the Kennedy Krieger Institute found that autopsy revealed inflammatory changes in the cerebellum with cytokine elevations in cortical areas and similar abnormal cytokine profiles in the cerebrospinal fluid of patients with ASD (16). However, the reason for this immune dysregulation remains unknown.

Most notably, T lymphocytes and monocytes have been implicated as immune system components involved with ASD. Another immune cell type, natural killer (NK) cells are also implicated (11, 13, 17–19). The human leukocyte antigen (HLA) system is the core of the development and maintenance of the immune system in humans. It is the most polymorphic of all human proteins, and specific polymorphisms have been demonstrated to have specific roles in the development of autoimmune disorders. For example, it is well known that the risk for developing ankylosing spondylitis is increased in individuals who have inherited HLA-B27. Thus, involvement of specific HLA components in the etiology of immune-associated ASD is plausible.

NK cells are thought to be most critical as part of the immune system’s first-line defense and immunosurveillance. Essentially, all the cells of the body (except for RBC) express HLA class I proteins. The expressed HLA-C locus components interact with NK cell killer immunoglobulin-like receptor (KIR) ligands. The HLA-C locus proteins are divided into two groups, group I or group II, based on the expression of 77 Ser and 80 Asn, in the sequence of the former, and expression 77 Asn and 80 Lys in the sequence of the latter. Further, HLA-C group I and group II reciprocally act as inhibitors and activators with specific KIR ligands expressed on the NK cells.

During normal immunosurveillance, an NK cell interrogating a human cell. If it finds the correct level of expression of HLA-C group I and group II components that “balance” activation and inhibition signaling *via* its KIR ligands, it leaves the cell unaffected. The normal ratio of expression of HLA-C group I to group II is 0.86. The presumption is that this was decided *via* evolution to provide the optimal NK cell immunosurveillance. Significant deviations from this may result in delayed immune system activation or perhaps over activation of immunity. For example, if the cell was infected by a virus, or was transformed and becoming cancerous, then the “normal” levels of HLA-C expression on the cell’s surface may be altered, and the NK cell may become activated *via* the KIR ligand interaction. This activation could generate inflammatory signals, and the NK cell may actively begin to kill the cell.

We hypothesized that a significant involvement of the immune system in ASD would be exhibited as bias in the distribution of specific HLA types. Further, we hypothesized that this would be occurring in a cohort of patients with similar clinical and laboratory features, rather than the entire population of ASD. To this end, we performed HLA typing on 126 patients with ASD and 17 lymphoblastoid cell lines (LCLs) derived from patients with ASD (143 total). We analyzed the typing results based on the normal expected frequencies for total and subpopulations of African-American, Asian, Caucasian, and Hispanic racial and ethnic distributions. We found that HLA-Cw7 was over-represented in patients with ASD, primarily in the Caucasian cohort. Further analyses for clinical and laboratory features indicate that those who express HLA-Cw7 have a higher rate of immune system–associated issues in comparison with those with ASD who do not express HLA-Cw7. Our data support the notion that the increased expression of HLA-Cw7 in a cohort of patients with ASD and immune system–associated issues may support chronic over-stimulation of NK cells, resulting in the phenotypic features of ASD.

## Methods

The study contains data derived from cell lines obtained from 126 participants diagnosed with ASD seen in at Arkansas Children’s Research Institute (Little Rock, AR) as part of two clinical studies (NCT02000284, NCT01602016) as well as 17 LCLs from the Autism Genetic Resource Exchange (AGRE) and the National Institutes of Mental Health biorepository. The primary purpose of the clinical studies from which these samples were derived was to investigate the relationship between mitochondrial dysfunction and ASD (NCT02000284) and abnormalities in folate metabolism and ASD (NCT01602016). Results on the primary studies from which these samples were derived has been published previously (20–28), but investigation of HLA types in relation to ASD has not been previously reported in these samples. Likewise, the LCLs used in this study have been used in previous laboratory studies examining mitochondrial dysfunction in relation to ASD (29–32), and the effect of environmental agents on mitochondrial (33–36) and immune (37) functions of the LCLs but investigation of the HLA types of these LCLs in relation to ASD has not been published previously. The Institutional Review Board (IRB) at the University of Arkansas for Medical Sciences (Little Rock, AR) approved the clinical studies and use of cell lines for the primary investigation and additional investigations such as HLA typing and other secondary genomic, metabolomic, and proteomic investigations. For clinical studies, parents of participants provided written informed consent. All experiments were performed in accordance with relevant guidelines and regulations.

### ASD Participants

One hundred twenty-six individuals with ASD met inclusion and exclusion criteria. **Table 1** outlines the clinical characteristics of these participants. Comorbid conditions were both derived from a parent reported medical questionnaire and from review of conditions diagnosed in the medical records. Regression (defined as loss of already obtained skills) was defined in detail in our questionnaire. Questions regarding regression included the timing, specific skills lost, duration of the regression, trigger, and whether multiple regressions were identified. Since this paper is not specifically on regression, we have not summarized these details in the table, only whether regression(s) were present or not present. This method for assessing medical comorbidities has been used in several of our previous studies (20, 21, 24, 25). We then selected the medical conditions that affected 10% or more of the individuals in the sample. Conditions with sample prevalence of less than 10% were considered unlikely to differentiate any immune subgroups defined by the HLA-typing analysis. Data for all the comorb+id conditions were not available for all participants, as some families did not complete the questionnaire or did not provide medical records for review, or the review of the medical records could not determine if the participant suffered from the comorbid condition or not. This information was missing at random and affected less than 10% of the participants. The total number of participants with the required information is outlined in the table below.

**Table 1:** ASD participant characteristics (N = 126).

Variable	
Age, mean (SD), years and months	7 years and 8 months (3 years and 6 months)
Males, N (%)	82% (22/126)
Caucasian	83% (104/126)
African American	7% (9/126)
Hispanic	3% (4/126)
Asian	7% (9/126)
**Comorbid conditions (parent report), % (N)**	
Abdominal pain	19% (23/118)
Allergies	43% (51/118)
Anxiety	26% (30/117)
ADHD	32% (37/117)
Sinusitis	13% (15/118)
Immune problems	14% (17/118)
Headaches	21% (25/118)
Regression	62% (72/116)
**Comorbid conditions (medical records), N (%)**	
Food allergies/intolerances	49% (56/115)
Chronic constipation	43% (49/113)
Chronic persistent infections	34% (41/119)
Immune disorder	10% (12/118)
Epilepsy	25% (29/118)

Inclusion criteria were (i) age 3 to 14 years of age and (ii) ASD diagnosis. Exclusion criteria were (i) chronic treatment with medications that would detrimentally affect mitochondrial function for the mitochondrial study from which these samples were derived, such as, antipsychotic medications, (ii) vitamin or mineral supplementation exceeding the recommended daily allowance, and (iii) prematurity.

The ASD diagnosis was defined by one of the following: (i) a gold-standard diagnostic instrument such as the Autism Diagnostic Observation Schedule (ADOS) and/or Autism Diagnostic Interview-Revised (ADI-R); (ii) the state of Arkansas diagnostic standard, defined as agreement of a physician, psychologist, and speech therapist; and/or (iii) DSM diagnosis by a physician along with standardized validated questionnaires and diagnosis confirmation by the Principal Investigator at the time (REF). We have validated that this criteria captures an accurate diagnosis of ASD in our previous studies by re-evaluating a portion of the participants with the ADI-R and determining that they all were well within the diagnostic criteria for ASD (20, 21, 24, 25).

### Cell Lines

As outlined in **Supplemental Table 1**, 17 LCLs were derived from white males diagnosed with autistic disorder with a pedigree having at least one other affected male sibling (i.e., multiplex family) (mean [SD] age 12.5 [3.0] years). These LCLs were obtained from the Autism Genetic Resource Exchange (AGRE; Los Angeles, CA, USA) and the National Institutes of Mental Health (NIMH; Bethesda, MD, USA) center for collaborative genomic studies on mental disorders. All relevant guidelines and regulations were followed. These de-identified human samples were determined to be exempt from IRB review. Boys from which these autistic disorder LCLs were derived were diagnosed with a gold-standard examination, either the ADOS or the ADI-R.

### DNA Isolation

DNA was isolated from buffy coats of blood collected in EDTA tubes using the Purgene DNA Purification Kit (Gentra Systems, Inc., Minneapolis, MN). For cell lines, DNA was isolated from 5x10^6^ pelleted cells using the same kit.

### HLA Typing

HLA typing was performed as previously reported using reagents and supplies from One Lambda^®^, in an American Society of Histocompatibility and Immunogenetics (ASHI) certified, and College of American Pathology (CAP) certified, laboratory using the LabType^®^ rSSO technique (38).

### Statistical Analyses

HLA-typing results for the patients and LCLs with ASD were compared with the distributions of HLA components reported by the United Network for Organ Sharing (UNOs) (https://unos.org/wp-content/uploads/unos/CPRA_frequencies.xls). These data are felt to reflect accurately the distributions of HLA types by race/ethnicity in the US. The racial/ethnic distribution in the specimens with ASD differs from those of the general population (**Supplemental Table 2**). Therefore, the expected frequency calculations were adjusted to accommodate the deviations by race/ethnicity from the general population. From 143 study subjects, 286 HLA alleles are present. The expected allele frequencies of the general population were determined by taking the fraction of each individual allele present and multiplying by 286, the total expected allele number. Thus, potential fractions of expected alleles are generated, and these are rounded to the nearest integer. Not all HLA types are found in the patients with ASD in our study. Thus, these cannot be analyzed, but the lack of these HLA types has potential to affect the expected values, since all HLA alleles at the serologic equivalence level were included in making the expected calculations. Therefore, the total numbers of expected alleles for each HLA-locus are projected not to completely add up to 286. Some HLA alleles are null alleles, and therefore not expressed. There were 2 HLA-C locus null alleles and 45 HLA-DR51/52/53 null alleles in the patients with ASD. Therefore, the total number of alleles in patients with ASD for HLA-C and HLA-DR51/52/53 were 284 and 241, respectively. HLA-DR51/52/53 were not analyzed separately by race ethnicity.

Chi-square analyses were performed for samples with larger numbers, and Fisher Exact Test analyses were performed with sample size approximately 20 or fewer. The p values were calculated using Microsoft Excel^®^, with p < 0.001 selected to represent the most stringent condition for determining an association with specific HLA types. Results were analyzed similar to previous studies searching for associations of HLA-typing results with disease (6–12, 13, 39).

To determine if the specific HLA subgroup identified was defined by specific medical symptoms, partial least squares discriminant analysis (PLSDA) using R version 3.5.0 mixOmics Omics Data Integration Project (version 6.3.2) was used to explore symptoms depicted in **Table 1** to determine if there were any specific symptoms that differentiated subgroups identified by the HLA-typing results.

## Results

### HLA Typing

HLA typing was performed on 143 total subjects (patients with ASD and LCLs from those diagnosed with ASD). The results were compared with the frequencies of individual HLA components from a very large national database (https://unos.org/wp-content/uploads/unos/CPRA_frequencies.xls), adjusted for the race/ethnic distributions of the subjects with ASD. The total group of patients was analyzed (see **Supplementary Tables 3–8**), and the HLA types were separately analyzed based on the individual race/ethnicity of the patients (see **Supplementary Tables 9–13**). Overall, HLA-Cw7 was found to be greatly over-represented from the expected in the total group and in the Caucasian cohort (p < 0.001) with significant positive association (**Table 2** and **Supplemental Tables 5 and 11**). HLA-DQ5 and HLA-DQ6 (**Supplementary Tables 8 and 13**) were nominally statistically significant but did not achieve statistical significance at the increased level we set at p < 0.001. HLA-A, HLA-B, HLA-DR, and HLA-DQ loci were not found to be represented with a significantly different frequency in ASD as compared to the general population (see **Supplementary Tables 3, 4, 6, 7, 9, 10, 12**). Likewise, loci on HLA-C and HLA-DQ other than those mentioned above were not found to be represented with a significantly different frequency in ASD as compared to the general population (see **Supplementary Tables 5, 8, 11, 13**).

**Table 2:** HLA-Cw7 is found more than expected in patients with ASD.

	ASD expected	ASD observed	p =
**Total ASD alleles = 286**	42	91	*0.000001
**Caucasian alleles = 242**	35	84	*0.0000002
**African American alleles = 18**	1	2	**0.3857
**Hispanic alleles = 8**	0	0	**1.0000
**Asian alleles = 18**	9	5	**0.1097

*p value by Chi Square, **p value by Fisher Exact Test.

The HLA types from specimens from 143 subjects with ASD were analyzed and compared with the expected distributions of HLA types in the normal population. Only HLA-Cw7 was found to be increased with respect to the expected distributions. Further, this was primarily due to more Caucasians with ASD expressing HLA-Cw7. Two hundred eighty-six alleles are present in 143 subjects. The expected allele frequency was calculated from the known distributions in the United States population and then adjusted for the frequencies of the race/ethnic frequencies in the ASD cohort. Samples with large numbers were analyzed by *chi square, and those with small sample numbers were analyzed by **Fischer Exact Test. P <0.001 was considered for statistical significance (**Supplemental Tables 2–13**).

In patients with ASD, HLA-C locus group II activating alleles are found more frequently than expected in the general population (**Table 3** and **Supplementary Table 5**). This increase in HLA-C locus activating alleles is primarily due to the increased frequency of expression of HLA-Cw7 (**Table 3**). The increase is due to the greater expression in Caucasians (**Table 4**; **Supplementary Table 11**).

**Table 3:** Increase in patients with ASD expressing HLA-Cw7 explains the increase in HLA-C group II activating alleles detected.

HLA-C	Total expected ASD	Group I activating	Group II activating	Total observed ASD	Group I activating	Group II activating	p =	HLA-C
1	10		10	10		10	*1.00	1
2	10	10		10	10		*1.00	2
4	32	32		32	32		**1.00	4
5	24	24		18	18		**0.86	5
6	24	24		25	25		**1.00	6
7	42		42	91		91	**0.0007	7
8	12		12	11		11	**1.00	8
9	19		19	19		19	**1.00	9
10	26		26	28		28	**1.00	10
12	16		16	17		17	**1.00	12
14	7		7	2		2	*0.44	14
15	8	8		5	5		*0.88	15
16	9	9		11	11		**0.98	16
17	5	5		3	3		*0.92	17
18	1	1		2	2		*0.95	18

*p value by Chi Square, **p value by Fisher Exact Test.

**Table 4:** Over-representation of HLA-C group II activating alleles observed in ASD.

	Total group I activating alleles	Total group II activating alleles	p =
**Total ASD**	95	189	*0.000002
**Caucasian**	73	167	*0.0000002
**African American**	9	9	**0.26
**Hispanic**	6	2	**0.06
**Asian**	7	11	**0.11

*p value from Chi Square, **p value from Fisher Exact Test.

The HLA-C locus components from subjects with ASD compared with the expected frequencies indicate an increase in group II Activating HLA-Cw7. All others are not statistically significantly different from the expected frequencies.

The total numbers of group I and group II activating alleles are compared by race/ethnicity in subjects with ASD. The total number of group II activating alleles is greater than expected in the total ASD cohort and the effect appears to be driven by Caucasians.

HLA-DQ5 and HLA-DQ6 were also found to trend toward being found in greater proportions in the subjects with ASD but did not achieve p < 0.001 statistical significance in this study (see **Supplementary Tables 8 and 13**). Inheritance of HLA-DQ6 has been previously associated with risk for scleroderma and narcolepsy. More subjects in a larger study may help to clarify the meaningfulness of this observation with regard to ASD.

### Clinical Characteristics of HLA Subgroups

One hundred eight participants had information on all clinical measures in order to enter into the PLSDA. Given the significant effect of the HLA-Cw7 allele, we divided the ASD participants in those that were negative for the HLA-Cw7 allele (N = 50) and those with at least one HLA-Cw7 allele (N = 58). The PLSDA found two components with the first and second components explaining 9 and 12% of the variance, respectively. The discriminant function demonstrated a 65% accuracy in discriminating the groups. The individual participant’s values on the two components are depicted in **Supplemental Figure 1**, and the correspondence of each clinical characteristic loading on the two components is given in **Supplemental Figure 2**.

**Figure 1:**
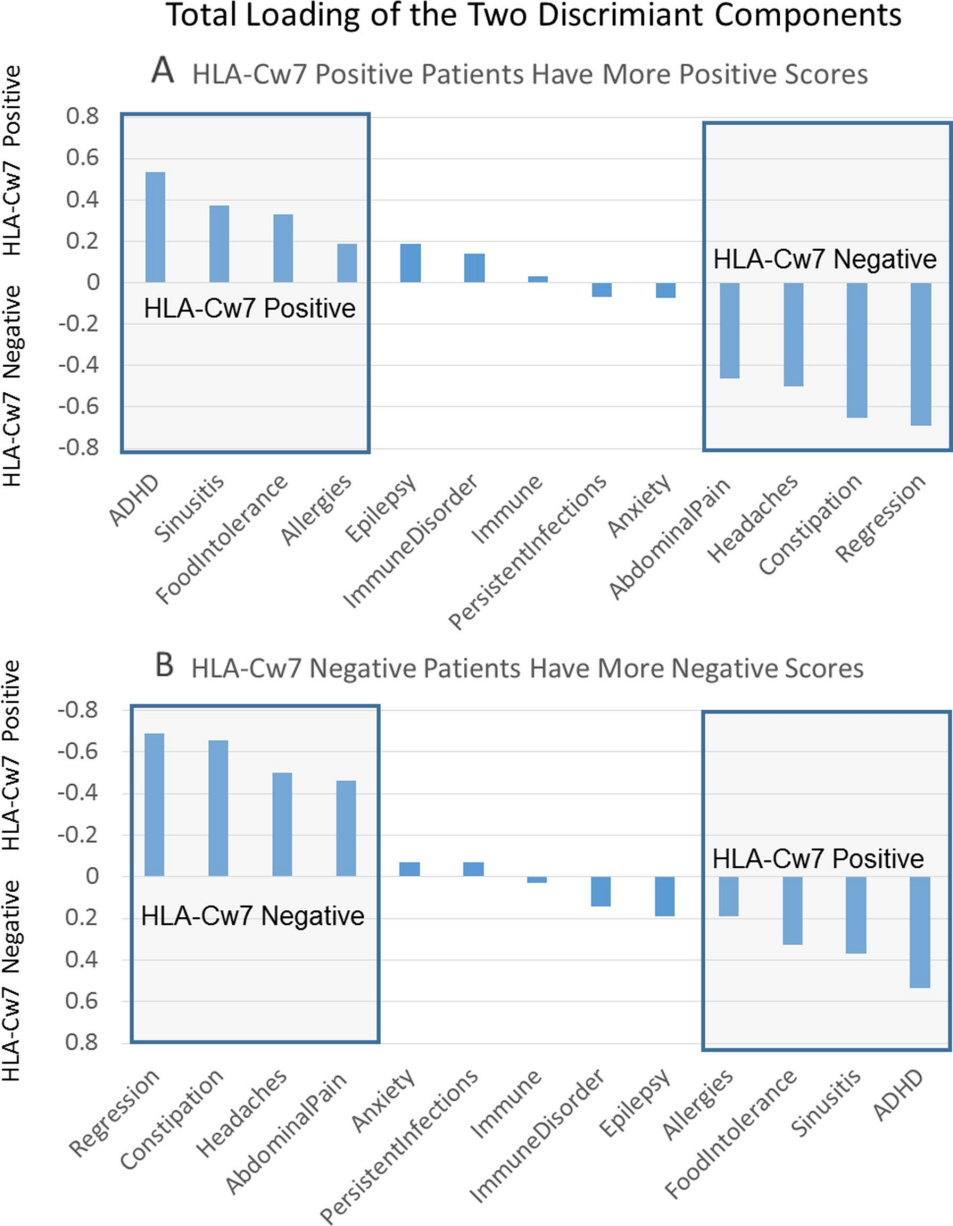
Total components of the discriminant function. **(A)** Positive numbers represent components that are related to HLA-Cw7-positive participants. **(B)** Negative numbers represent components more representative of the HLA-Cw7-negative participants.

**Supplemental Table 15** outlines the loading for the two individual components of the discriminant function as well as the total loading for both components. This is sorted by the loading value for each clinical characteristic and presented graphically in **Figure 1**. Positive loadings represent characteristics that are more representative of HLA-Cw7-positive participants whereas negative loading represent characteristics more representative of the HLA-Cw7-negative participants. Considering the top loadings for those who are HLA-Cw7-positive, we find that two of the three characteristics of HLA-Cw7-positive participants in our sample are related to immune system activation, including allergies, food intolerances, and chronic sinusitis, whereas none of the characteristics of the HLA-Cw7-negative patients involve immune system activation.

**Figure 2:**
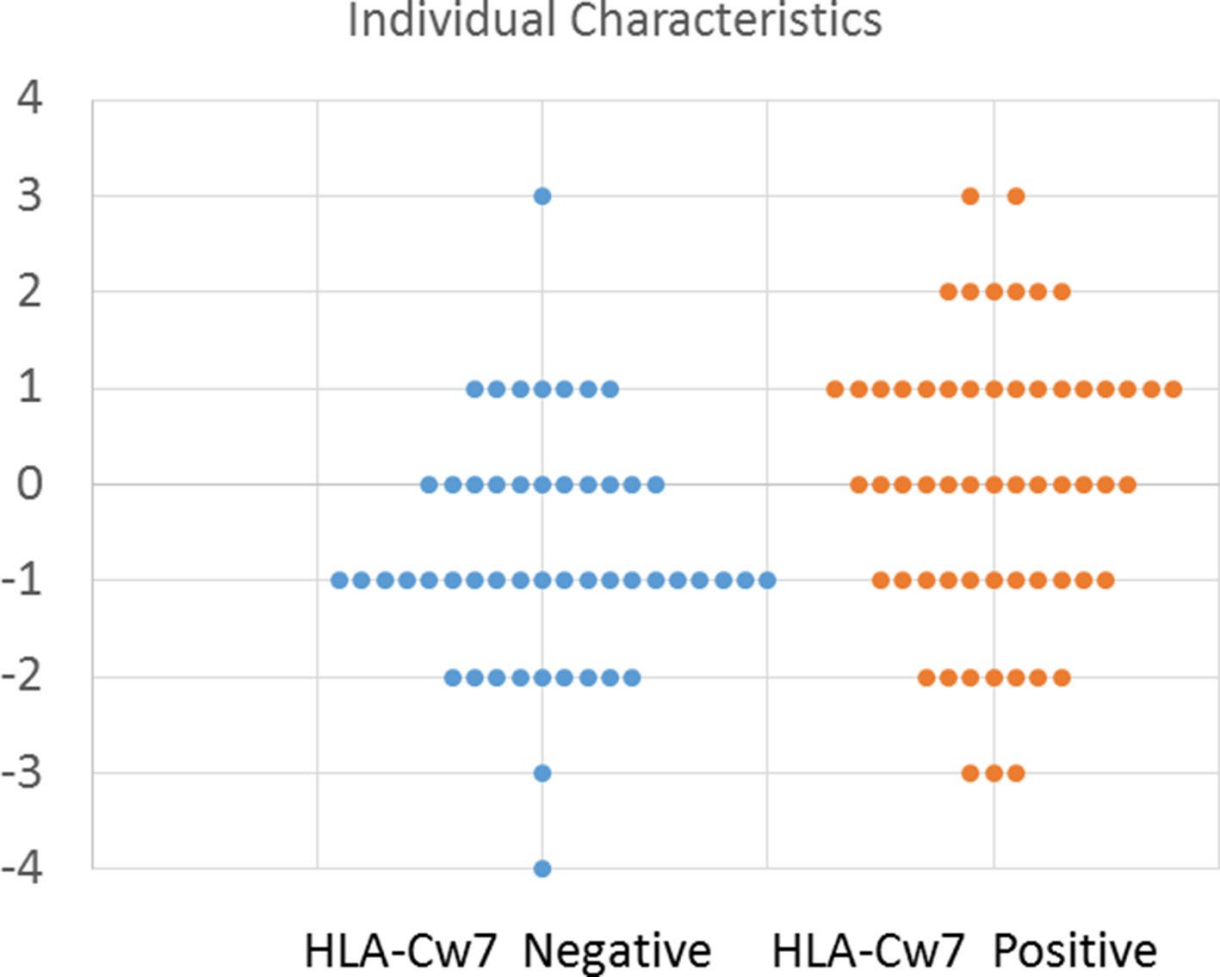
Number of characteristics for HLA-Cw7-negative and HLA-Cw7-positive participants. Clinical characteristics that are more representative of absence of HLA-Cw7 indicate weighted negative, and clinical characteristics of presence of HLA-Cw7 indicate weighted positive.

To better depict the characteristics of individual participants, we created an index to describe the number of clinical symptoms attributed to HLA-Cw7-negative and HLA-Cw7-positive participants in the analysis. A value of −1 was assigned for each of the top four clinical characteristics of HLA-Cw7-negative participants (regression, chronic constipation, headaches, abdominal pain), and a value of +1 was assigned for each of the top four clinical characteristics of HLA-Cw7-positive participants (allergies, food intolerances sinusitis, ADHD). As seen in **Figure 2**, HLA-Cw7-positive participants demonstrated a more positive score, on average, but also were more variable in their characteristics.

## Discussion

The specific etiologies responsible for causing ASD remain to be fully determined. Patients with ASD, while having an overall similar neuropsychiatric phenotype resulting in the diagnosis, have quite a variability in other clinical features and laboratory studies. It is expected that the individual’s genetics influenced by environmental exposures results in the disease.

Many factors have implicated the immune system involvement in ASD. Notably, inflammatory effects on the central nervous system incriminate some alteration in immune function or dysregulation of immunity (14). Identification of specific cell types and targets, as can be found in specific autoimmune disorders, has not been rewarding at this point in time. Further, it is doubtful that one specific immune dysfunctional cell type and target are responsible for all ASD. It is reasonable and plausible that specific cohorts of individuals with ASD united by similar clinical and laboratory features may have similar underlying forms of immune dysfunction resulting in some form of chronic inflammation, which results in the neurologic features of disease. Since ASD tends to occur early in life, the genetic features and environmental exposures would have to be part of this early presentation. Further, since HLA components are frequently found associated with specific autoimmune disorders, are expressed on the body’s cells from the onset, and shape the immune system maturation and function, it is reasonable that specific HLA components could result in the immune system’s response in such an adverse manner.

Previous studies have implicated specific HLA types to be associated with ASD (6–13). Perhaps, HLA-DR4 has been most commonly found in association (7, 8, 10, 11). HLA-A2 has also been commonly reported (6, 9, 10). Specific HLA haplotypes have been reported (HLA- A*01-B*07-DRB1*0701-DQB1*0602) in patients from Saudi Arabia, and HLA-A2-B44-DR4 and HLA-B44-C30-DR4, from different studies in the United States (10–12). At the stringent p value of p < 0.001, we were unable to detect any of these associations (**Supplemental Tables 3–13**). From these reports, it may be ascertained that different geographic locations of patients with ASD may had predilection for different HLA type preferences. Although not specifically studied or stated in these reports, post-streptococcal immunoreactivity is correlated with HLA-DR4, and it is unknown whether PANDAS could be having a role in the bias toward finding an association with HLA-DR4 in some of the studies. Further, studies have not specifically sought for HLA-C locus associations, until the present study. Additionally, other studies have not specifically adjusted the expected control group HLA frequencies to the race/ethnic frequencies found in the ASD subjects. We believe that the careful adjustment for the race/ethnic distributions of the HLA types between the control/expected HLA distributions and the observed HLA types in our subjects with ASD has resulted in the difference found in our study *versus* those previously reported.

We found that a cohort of patients with ASD (primarily Caucasian) expresses HLA-Cw7 at more than twice the extent expected in the normal population. These patients share immune-associated clinical features, not found in patients who do not express HLA-Cw7. Interestingly, others have associated a subset of individuals with ASD and non-IgE-mediated food allergy (40) and chronic sinusitis (41) who have dysregulation in cytokine production and microRNA expression and dysregulation of innate immunity, although HLA distribution has not been studied in this subset. While HLA-DQ5 and HLA-DQ6 trended toward greater presence in subjects with ASD, since statistical significance was not achieved, we await further analyses with a larger number of subjects with ASD to pursue further the potential meaningfulness of these findings.

The HLA-C locus is expressed on all cell types (except RBC) and has a major role in immunosurveillance. HLA-C interacts with NK cell KIR ligands in an activating and inhibiting fashion. Thus, when an NK cell interrogates a cell in the body, the correct expression of HLA-C on the body cell prevents the NK cell from becoming activated. When a cell becomes infected with a virus, one of the viral strategies to prevent recognition is to decrease the expression of HLA on the cell surface. In doing so, the alteration in HLA-C expression can alert NK cells to attack. Cancerous cells are thought to be targeted by NK cells in a similar fashion, i.e., alteration in the normal levels of HLA-C expression.

The increased frequency of subjects with HLA-Cw7 expression also results in these subjects having an overall greater expression of the HLA-C group II activating alleles. While it remains to be proven, we suspect this cohort of patients may have a continuous and ongoing level of inflammation generated by NK cells in response to this increase in group II activating alleles. This could result in the overall increase in immune-associated clinical features in this cohort. Warren et al. (17) first investigated NK cell cytotoxic activity in ASD in 1987 and found reduced *ex vivo* NK cell cytotoxic activity in 12/31 ASD subjects examined. This finding was supported by Vojdani et al. (18) who demonstrated reduced *ex vivo* NK cell cytotoxic activity in 45% of ASD children in a much larger cohort (1,027 ASD subjects). Shortly thereafter, Enstrom et al. (19) demonstrated increased numbers of NK cells as well as upregulation of NK cell receptors and cytolytic effector molecules in 52 children with ASD as compared to 27 controls. Importantly, while *ex vivo* NK cell cytotoxic activity was reduced in the ASD cohort, interferon-gamma-producing NK cells were increased under resting conditions in children with ASD suggesting that NK cells in the ASD cohort are maximally activated *in vivo* and, thus, chronically activated NK cells are down-regulated when stimulated *ex vivo*. Unfortunately, HLA typing was not performed in these three studies. Taken together, these data support the notion that a subset of ASD subjects with greater expression of HLA-C group II activating alleles may have chronically activated NK cells. Torres et al. have previously reported the association of activating KIR ligands and ASD (11, 13). Indeed, the KIR-activating gene 2DS1 was shown to be increased in frequency in patients with ASD, and this supports our findings as HLA-Cw7 is a cognate ligand for KIR 2DS1 (13). Thus, this could result in a greater level of NK cell activation in patients with ASD who express HLA-Cw7.

Further studies in patients with ASD who express HLA-Cw7 are needed with specific evaluation of NK cell activation *in vivo*. This could potentially lead to therapeutic intervention by specifically targeting over-active NK cells in patients with ASD who express HLA-Cw7. The overall decrease in chronic inflammation could then decrease the burden on the central nervous system in a cohort of patients with ASD.

## Ethics Statement

The Institutional Review Board (IRB) at the University of Arkansas for Medical Sciences (Little Rock, AR) approved the clinical studies and use of cell lines. For clinical studies, parents of participants provided written informed consent. All experiments were performed in accordance with relevant guidelines and regulations.

## Author Contributions

All authors were involved in the design and conceptualization of the experiments. Laboratory experiments were conducted by TH, BR-C, SB, RW and SR. LD, JS, MT and RF were involved in subject recruitment and assessment. Data was analyzed by TH, LD, SR, SK and RF. All authors were involved in drafting, editing and finalizing the manuscript. All authors approved of the final manuscript.

## Funding

We would like to thank the supporters of our research studies, including the Arkansas Biosciences Institute (Little Rock, AR, USA), the Jonty Foundation (St. Paul, MN), the Autism Research Institute (San Diego, CA), the Gupta Family Foundation (Atherton, CA), the Jane Bostford Johnson Foundation (New York, NY), the Jager Family Foundation (Chicago, IL) and the Phoenix Children’s Hospital Foundation (Phoenix, AZ). HLA typing was in part funded by Department of Pathology, UAMS, Research Funds.

## Conflict of Interest Statement

JS is the Co-Founder, President and Chief Executive Officer of BioRosa Technologies Inc, a start-up company focused on developing a diagnostic test for ASD based on metabolic biomarkers. JS contributed in the collection of participant data during his employment at the University of Arkansas for Medical Sciences as the clinical trials manager for the autism research program at Arkansas Children’s Research Institute. No data or samples were shared with JS or BioRosa for commerical use.

The remaining authors declare that the research was conducted in the absence of any commercial or financial relationships that could be construed as a potential conflict of interest.
