# Supplementary material for: Inheritance of HLA-Cw7 Associated With Autism Spectrum Disorder (ASD)
Source: Front Psychiatry. 2019 Sep 11;10:612. doi: 10.3389/fpsyt.2019.00612 (PMC6749146; doi:10.3389/fpsyt.2019.00612)
Supplement: Supplementary file 1 [file DataSheet_1.docx]

| **Supplementary Table 1. Lymphoblastoid cell lines examined in the current study** | | | | | |
| --- | --- | --- | --- | --- | --- |
| **Cell ID** | **Source** | **Age (y)** | **Cell ID** | **Source** | **Age (y)** |
| 02C10618 | NIMH | 7 | 03C14441 | NIMH | 7 |
| AU1165302 | AGRE | 13 | 03C14349 | NIMH | 17 |
| AU0939303 | AGRE | 11 | 03C17237 | NIMH | 10 |
| AU1393306 | AGRE | 3 | AU038804 | AGRE | 8 |
| 05C38988 | NIMH | 12 | 03C15992 | NIMH | 5 |
| 01C08022 | NIMH | 5 | 01C08495 | NIMH | 4 |
| 03C16499 | NIMH | 11 | 02C09713 | NIMH | 7 |
| 01C08367 | NIMH | 7 | 01C08594 | NIMH | 7 |
| AU1215301 | AGRE | 12 | 04C26296 | NIMH | 10 |
| 00C04757 | NIMH | 10 | 04C27439 | NIMH | 7 |
| 02C09650 | NIMH | 7 | AU008404 | AGRE | 13 |
| 02C10054 | NIMH | 6 | 03C14363 | NIMH | 4 |
| AU1267302 | AGRE | 10 |  |  |  |

Coriell = Coriell Cell Repository (Camden, NJ, USA); NIMH = National Institutes of Mental Health Biorepository (Bethesda, MD, USA); AGRE= Autism Genetic Resource Exchange Biorepository (Los Angeles, CA, USA)

Supplemental Table 2. The expected distributions by race/ethnicity from a large HLA database (<https://bioinformatics.bethematchclinical.org/hla-resources/haplotype-frequencies/high-resolution-hla-alleles-and-haplotypes-in-the-us-population/>) with the observed frequencies from out study population are shown.

|  | **Expected** | **Observed** |
| --- | --- | --- |
| **Caucasian** | **0.687** | **0.832** |
| **African American** | **0.147** | **0.071** |
| **Hispanic** | **0.143** | **0.033** |
| **Asian** | **0.023** | **0.054** |

Supplemental Table 3. HLA-A Locus Distributions of Patients with ASD are not Statistically Significantly Different from the Expected Distributions. One hundred forty three subjects with ASD were studied, providing 286 HLA alleles. The expected ASD number for the HLA-A locus allele is calculated by adjusting the known race/ethnic frequency of the specific HLA allele with the frequency found in the ASD study population. The observed is the actual number of occurrences of the specific allele. Chi square analysis was used to determine whether any allele was found in higher of lower proportions from the expected, with significance set at p<0.001. There were no statistically significant differences among the HLA-A locus alleles.

| **A Locus** | **Expected ASD Number** | **ASD Observed Number** | **Chi Square** | **p =** | **A Locus** |
| --- | --- | --- | --- | --- | --- |
| **1** | **40** | **41** | **0.0145** | **0.9042** | **1** |
| **2** | **81** | **80** | **0.0087** | **0.9256** | **2** |
| **3** | **36** | **41** | **0.3782** | **0.5385** | **3** |
| **11** | **19** | **18** | **0.0291** | **0.8645** | **11** |
| **23** | **8** | **10** | **0.2311** | **0.6307** | **23** |
| **24** | **26** | **26** | **0.0000** | **1.0000** | **24** |
| **25** | **5** | **6** | **0.0934** | **0.7600** | **25** |
| **26** | **9** | **10** | **0.0548** | **0.8149** | **26** |
| **29** | **11** | **11** | **0.0000** | **1.0000** | **29** |
| **30** | **9** | **5** | **1.1800** | **0.2774** | **30** |
| **31** | **8** | **4** | **1.3717** | **0.2415** | **31** |
| **32** | **9** | **10** | **0.0548** | **0.8149** | **32** |
| **33** | **6** | **4** | **0.4100** | **0.5220** | **33** |
| **34** | **2** | **2** | **0.0000** | **1.0000** | **34** |
| **66** | **1** | **2** | **0.3375** | **0.5613** | **66** |
| **68** | **9** | **15** | **1.5772** | **0.2092** | **68** |
| **74** | **1** | **1** | **0.0000** | **1.0000** | **74** |

Supplemental Table 4. HLA-B Locus Distributions of Patients with ASD are not Statistically Significantly Different from the Expected Distributions. One hundred forty three subjects with ASD were studied, providing 286 HLA alleles. The expected ASD number for the HLA-B locus allele is calculated by adjusting the known race/ethnic frequency of the specific HLA allele with the frequency found in the ASD study population. The observed is the actual number of occurrences of the specific allele. Chi square analysis was used to determine whether any allele was found in higher of lower proportions from the expected, with significance set at p<0.001. There were no statistically significant differences among the HLA-B locus alleles.

| **B Locus** | **Expected ASD Number** | **ASD Observed Number** | **Chi Square** | **p =** | **B Locus** |
| --- | --- | --- | --- | --- | --- |
| **7** | **35** | **40** | **0.3867** | **0.5340** | **7** |
| **8** | **28** | **25** | **0.1886** | **0.6641** | **8** |
| **13** | **7** | **3** | **1.6401** | **0.2003** | **13** |
| **18** | **13** | **8** | **1.2449** | **0.2645** | **18** |
| **27** | **11** | **13** | **0.1752** | **0.6755** | **27** |
| **35** | **25** | **27** | **0.0853** | **0.7703** | **35** |
| **37** | **3** | **7** | **1.6401** | **0.2003** | **37** |
| **38** | **5** | **5** | **0.0000** | **1.0000** | **38** |
| **39** | **5** | **9** | **1.1800** | **0.2774** | **39** |
| **41** | **2** | **4** | **0.6785** | **0.4101** | **41** |
| **44** | **39** | **38** | **0.0151** | **0.9021** | **44** |
| **45** | **3** | **1** | **1.0142** | **0.3139** | **45** |
| **46** | **1** | **1** | **0.0000** | **1.0000** | **46** |
| **47** | **1** | **1** | **0.0000** | **1.0000** | **47** |
| **48** | **1** | **2** | **0.3375** | **0.5613** | **48** |
| **49** | **4** | **7** | **0.8402** | **0.3593** | **49** |
| **51** | **14** | **11** | **0.3792** | **0.5380** | **51** |
| **52** | **3** | **2** | **0.2032** | **0.6522** | **52** |
| **55** | **5** | **7** | **0.3429** | **0.5581** | **55** |
| **56** | **2** | **3** | **0.2032** | **0.6522** | **56** |
| **57** | **10** | **11** | **0.0498** | **0.8234** | **57** |
| **58** | **4** | **5** | **0.1137** | **0.7360** | **58** |
| **60** | **15** | **16** | **0.0344** | **0.8529** | **60** |
| **61** | **5** | **3** | **0.5107** | **0.4748** | **61** |
| **62** | **17** | **19** | **0.1195** | **0.7296** | **62** |
| **64** | **1** | **4** | **1.8288** | **0.1763** | **64** |
| **65** | **4** | **6** | **0.4100** | **0.5220** | **65** |
| **70** | **2** | **1** | **0.3375** | **0.5613** | **70** |
| **71** | **1** | **3** | **1.0142** | **0.3139** | **71** |
| **72** | **1** | **1** | **0.0000** | **1.0000** | **72** |
| **73** | **0** | **1** | **1.0088** | **0.3152** | **73** |
| **81** | **0** | **2** | **2.0212** | **0.1551** | **81** |

Supplemental Table 5. HLA-C Locus Distributions of Patients with ASD Indicate an Increased Presence of HLA-Cw7. One hundred forty three subjects with ASD were studied, providing 286 HLA alleles, but two null alleles were present. The expected ASD number for the HLA-C locus allele is calculated by adjusting the known race/ethnic frequency of the specific HLA allele with the frequency found in the ASD study population. The observed is the actual number of occurrences of the specific allele. Chi square analysis was used to determine whether any allele was found in higher of lower proportions from the expected, with significance set at p<0.001. HLA-Cw7 was found to be statistically significantly found expressed on a greater number of subjects with ASD.

| **C Locus** | **Expected ASD Number** | **ASD Observed Number** | **Chi Square** | **p =** | **C Locus** |
| --- | --- | --- | --- | --- | --- |
| **1** | **10** | **10** | **0.0000** | **1.0000** | **1** |
| **2** | **10** | **10** | **0.0000** | **1.0000** | **2** |
| **4** | **32** | **32** | **0.0000** | **1.0000** | **4** |
| **5** | **24** | **18** | **0.9321** | **0.3343** | **5** |
| **6** | **24** | **25** | **0.0225** | **0.8808** | **6** |
| **7** | **42** | **91** | **23.7382** | **0.000001** | **7** |
| **8** | **12** | **11** | **0.0456** | **0.8308** | **8** |
| **9** | **19** | **19** | **0.0000** | **1.0000** | **9** |
| **10** | **26** | **28** | **0.0824** | **0.7740** | **10** |
| **12** | **16** | **17** | **0.0324** | **0.8572** | **12** |
| **14** | **7** | **2** | **2.8424** | **0.0918** | **14** |
| **15** | **8** | **5** | **0.7135** | **0.3983** | **15** |
| **16** | **9** | **11** | **0.2088** | **0.6477** | **16** |
| **17** | **5** | **3** | **0.5107** | **0.4748** | **17** |
| **18** | **1** | **2** | **0.3375** | **0.5613** | **18** |

Supplemental Table 6. HLA-DR Locus Distributions of Patients with ASD are not Statistically Significantly Different from the Expected Distributions. One hundred forty three subjects with ASD were studied, providing 286 HLA alleles. The expected ASD number for the HLA-DR locus allele is calculated by adjusting the known race/ethnic frequency of the specific HLA allele with the frequency found in the ASD study population. The observed is the actual number of occurrences of the specific allele. Chi square analysis was used to determine whether any allele was found in higher of lower proportions from the expected, with significance set at p<0.001. There were no statistically significant differences among the HLA-DR locus alleles.

| **DR Locus** | **Expected ASD Number** | **ASD Observed Number** | **Chi Square** | **p =** | **DR Locus** |
| --- | --- | --- | --- | --- | --- |
| **1** | **27** | **37** | **1.7733** | **0.1830** | **1** |
| **4** | **46** | **39** | **0.6827** | **0.4087** | **4** |
| **7** | **35** | **36** | **0.0162** | **0.8987** | **7** |
| **8** | **11** | **10** | **0.0498** | **0.8234** | **8** |
| **9** | **5** | **8** | **0.7135** | **0.3983** | **9** |
| **10** | **3** | **5** | **0.5107** | **0.4748** | **10** |
| **11** | **27** | **33** | **0.6756** | **0.4111** | **11** |
| **12** | **7** | **5** | **0.3429** | **0.5581** | **12** |
| **13** | **33** | **32** | **0.0175** | **0.8948** | **13** |
| **14** | **8** | **9** | **0.0611** | **0.8048** | **14** |
| **15** | **40** | **42** | **0.0574** | **0.8106** | **15** |
| **16** | **4** | **6** | **0.4100** | **0.5220** | **16** |
| **17** | **25** | **24** | **0.0225** | **0.8808** | **17** |

Supplemental Table 7. HLA-DRw Locus Distributions of Patients with ASD are not Statistically

Significantly Different from the Expected Distributions. One hundred forty three subjects with ASD were studied, providing 286 HLA alleles, there were forty five null alleles in the ASD subjects. The expected ASD number for the HLA-DRw locus allele is calculated by adjusting the known race/ethnic frequency of the specific HLA allele with the frequency found in the ASD study population. The observed is the actual number of occurrences of the specific allele. Chi square analysis was used to determine whether any allele was found in higher of lower proportions from the expected, with significance set at p<0.001. There were no statistically significant differences among the HLA-DRw locus alleles.

| **DRw Locus** | **Expected ASD Number** | **ASD Observed Number** | **Chi Square** | **p =** | **DRw Locus** |
| --- | --- | --- | --- | --- | --- |
| **51** | **46** | **46** | **0.0000** | **1.0000** | **51** |
| **52** | **104** | **104** | **0.0000** | **1.0000** | **52** |
| **53** | **71** | **71** | **0.0000** | **1.0000** | **53** |

Supplemental Table 8. HLA-DQ Locus Distributions of Patients with ASD are not Statistically Significantly Different from the Expected Distributions. One hundred forty three subjects with ASD were studied, providing 286 HLA alleles. The expected ASD number for the HLA-DQ locus allele is calculated by adjusting the known race/ethnic frequency of the specific HLA allele with the frequency found in the ASD study population. The observed is the actual number of occurrences of the specific allele. Chi square analysis was used to determine whether any allele was found in higher of lower proportions from the expected, with significance set at p<0.001. There were no statistically significant differences among the HLA-DQ locus alleles, although HLA-DQ5 and HLA-DQ6 approached statistical significance.

| **DQ Locus** | **Expected ASD Number** | **ASD Observed Number** | **Chi Square** | **p =** | **DQ Locus** |
| --- | --- | --- | --- | --- | --- |
| **2** | **59** | **48** | **1.4031** | **0.2362** | **2** |
| **4** | **10** | **9** | **0.0548** | **0.8149** | **4** |
| **5** | **29** | **55** | **9.5108** | **0.0020** | **5** |
| **6** | **43** | **74** | **10.4173** | **0.0012** | **6** |
| **7** | **41** | **57** | **3.1792** | **0.0746** | **7** |
| **8** | **16** | **21** | **0.7278** | **0.3936** | **8** |
| **9** | **14** | **22** | **1.9114** | **0.1668** | **9** |

Supplemental Table 9. HLA-A Locus Distributions by Race/Ethnicity of Patients with ASD are not Statistically Significantly Different from the Expected Distributions. One hundred forty three subjects with ASD were studied, providing 286 HLA alleles. The expected ASD number for the HLA-A locus allele is calculated by adjusting the known race/ethnic frequency of the specific HLA allele with the frequency found in the ASD study population. This was then further divided into specific race/ethic groups, providing 242 alleles in Caucasians, 18 alleles in African Americans, 8 alleles in Hispanics, and 18 alleles in Asians with ASD. The observed is the actual number of occurrences of the specific allele. *Chi square analysis was used to determine whether any allele was found in higher of lower proportions from the expected for Caucasians, and **Fisher Exact test was used for the remainder, with significance set at p<0.001. There were no statistically significant differences among the HLA-A locus alleles.

|  | **Caucasian alleles=242** | | | |  | **African American alleles=18** | | |  | **Hispanic alleles=8** | | |  | **Asian alleles=18** | | |
| --- | --- | --- | --- | --- | --- | --- | --- | --- | --- | --- | --- | --- | --- | --- | --- | --- |
| **A**  **Locus** | **ASD Exp** | **ASD Obs** | **Chi Sq** | ***p =** |  | **ASD Exp** | **ASD Obs** | ****p =** |  | **ASD Exp** | **ASD Obs** | ****p =** |  | **ASD Exp** | **ASD Obs** | ****p =** |
| **1** | **38** | **35** | **0.1452** | **0.7032** |  | **0** | **1** | **0.5000** |  | **0** | **2** | **0.2333** |  | **1** | **3** | **0.2494** |
| **2** | **71** | **72** | **0.0099** | **0.9206** |  | **1** | **3** | **0.2494** |  | **0** | **2** | **0.2333** |  | **11** | **3** | **0.0068** |
| **3** | **33** | **38** | **0.4126** | **0.5206** |  | **1** | **0** | **0.5000** |  | **0** | **2** | **0.2333** |  | **1** | **1** | **0.5143** |
| **11** | **15** | **15** | **0.0000** | **1.0000** |  | **0** | **0** | **1.0000** |  | **0** | **1** | **0.5000** |  | **7** | **2** | **0.0517** |
| **23** | **5** | **6** | **0.0930** | **0.7604** |  | **1** | **3** | **0.2494** |  | **0** | **0** | **1.0000** |  | **0** | **1** | **0.5000** |
| **24** | **20** | **18** | **0.1142** | **0.7354** |  | **0** | **4** | **0.0519** |  | **0** | **1** | **0.5000** |  | **8** | **3** | **0.0594** |
| **25** | **5** | **6** | **0.0930** | **0.7604** |  | **0** | **0** | **1.0000** |  | **0** | **0** | **1.0000** |  | **0** | **0** | **1.0000** |
| **26** | **7** | **8** | **0.0688** | **0.7931** |  | **0** | **0** | **1.0000** |  | **0** | **0** | **1.0000** |  | **2** | **2** | **0.3974** |
| **29** | **9** | **10** | **0.0548** | **0.8149** |  | **0** | **1** | **0.5000** |  | **0** | **0** | **1.0000** |  | **0** | **0** | **1.0000** |
| **30** | **6** | **5** | **0.0930** | **0.7604** |  | **1** | **0** | **0.5000** |  | **0** | **0** | **1.0000** |  | **1** | **0** | **0.5000** |
| **31** | **7** | **4** | **0.8372** | **0.3602** |  | **0** | **0** | **1.0000** |  | **0** | **0** | **1.0000** |  | **1** | **0** | **0.5000** |
| **32** | **9** | **8** | **0.0610** | **0.8050** |  | **0** | **1** | **0.5000** |  | **0** | **0** | **1.0000** |  | **0** | **1** | **0.5000** |
| **33** | **3** | **3** | **0.0000** | **1.0000** |  | **1** | **0** | **0.5000** |  | **0** | **0** | **1.0000** |  | **4** | **1** | **0.1461** |
| **34** | **0** | **0** | **0.1000** | **0.7518** |  | **0** | **2** | **0.2429** |  | **0** | **0** | **1.0000** |  | **1** | **0** | **0.5000** |
| **66** | **1** | **2** | **0.3354** | **0.5625** |  | **0** | **0** | **1.0000** |  | **0** | **0** | **1.0000** |  | **0** | **0** | **1.0000** |
| **68** | **7** | **12** | **1.3696** | **0.2419** |  | **0** | **2** | **0.2429** |  | **0** | **0** | **1.0000** |  | **1** | **1** | **0.5143** |
| **74** | **0** | **0** | **0.1000** | **0.7518** |  | **0** | **1** | **0.5000** |  | **0** | **0** | **1.0000** |  | **0** | **0** | **1.0000** |

Supplemental Table 10. HLA-B Locus Distributions by Race/Ethnicity of Patients with ASD are not Statistically Significantly Different from the Expected Distributions. One hundred forty three subjects with ASD were studied, providing 286 HLA alleles. The expected ASD number for the HLA-B locus allele is calculated by adjusting the known race/ethnic frequency of the specific HLA allele with the frequency found in the ASD study population. This was then further divided into specific race/ethic groups, providing 242 alleles in Caucasians, 18 alleles in African Americans, 8 alleles in Hispanics, and 18 alleles in Asians with ASD. The observed is the actual number of occurrences of the specific allele. *Chi square analysis was used to determine whether any allele was found in higher of lower proportions from the expected for Caucasians, and **Fisher Exact test was used for the remainder, with significance set at p<0.001. There were no statistically significant differences among the HLA-B locus alleles.

|  | **Caucasian alleles=242** | | | |  | **African American alleles=18** | | |  | **Hispanic alleles=8** | | |  | **Asian alleles=18** | | |
| --- | --- | --- | --- | --- | --- | --- | --- | --- | --- | --- | --- | --- | --- | --- | --- | --- |
| **B**  **Locus** | **ASD Exp** | **ASD Obs** | **Chi Sq** | ***p =** |  | **ASD Exp** | **ASD Obs** | ****p =** |  | **ASD Exp** | **ASD Obs** | ****p =** |  | **ASD Exp** | **ASD Obs** | ****p =** |
| **7** | **32** | **36** | **0.2738** | **0.6008** |  | **0** | **1** | **0.5000** |  | **0** | **0** | **1.0000** |  | **2** | **3** | **0.3312** |
| **8** | **27** | **24** | **0.1973** | **0.6569** |  | **0** | **1** | **0.5000** |  | **0** | **0** | **1.0000** |  | **0** | **0** | **1.0000** |
| **13** | **6** | **3** | **1.0189** | **0.3128** |  | **0** | **0** | **1.0000** |  | **0** | **0** | **1.0000** |  | **2** | **0** | **0.2429** |
| **18** | **11** | **6** | **1.5241** | **0.2170** |  | **0** | **1** | **0.5000** |  | **0** | **0** | **1.0000** |  | **1** | **1** | **0.5143** |
| **27** | **10** | **12** | **0.1905** | **0.6625** |  | **0** | **1** | **0.5000** |  | **0** | **0** | **1.0000** |  | **1** | **0** | **0.5000** |
| **35** | **21** | **21** | **0.0000** | **1.0000** |  | **0** | **2** | **0.2429** |  | **0** | **1** | **0.5000** |  | **4** | **3** | **0.2991** |
| **37** | **3** | **5** | **0.5084** | **0.4758** |  | **0** | **0** | **1.0000** |  | **0** | **1** | **0.5000** |  | **0** | **1** | **0.5000** |
| **38** | **4** | **5** | **0.1132** | **0.7365** |  | **0** | **0** | **1.0000** |  | **0** | **0** | **1.0000** |  | **2** | **0** | **0.2429** |
| **39** | **4** | **9** | **1.9762** | **0.1598** |  | **0** | **0** | **1.0000** |  | **0** | **0** | **1.0000** |  | **1** | **0** | **0.5000** |
| **41** | **2** | **4** | **0.6750** | **0.4113** |  | **0** | **0** | **1.0000** |  | **0** | **0** | **1.0000** |  | **0** | **0** | **1.0000** |
| **44** | **36** | **32** | **0.2738** | **0.6008** |  | **0** | **2** | **0.2429** |  | **0** | **3** | **0.1000** |  | **2** | **1** | **0.3857** |
| **45** | **2** | **1** | **0.3354** | **0.5625** |  | **0** | **0** | **1.0000** |  | **0** | **0** | **1.0000** |  | **0** | **0** | **1.0000** |
| **46** | **0** | **0** | **0.1000** | **0.7518** |  | **0** | **1** | **0.5000** |  | **0** | **0** | **1.0000** |  | **2** | **0** | **0.2429** |
| **47** | **1** | **1** | **0.0000** | **1.0000** |  | **0** | **0** | **1.0000** |  | **0** | **0** | **1.0000** |  | **0** | **0** | **1.0000** |
| **48** | **0** | **1** | **1.0021** | **0.3168** |  | **0** | **0** | **1.0000** |  | **0** | **1** | **0.5000** |  | **1** | **0** | **0.5000** |
| **49** | **4** | **7** | **0.8372** | **0.3602** |  | **0** | **0** | **1.0000** |  | **0** | **0** | **1.0000** |  | **0** | **0** | **1.0000** |
| **51** | **12** | **11** | **0.0456** | **0.8308** |  | **0** | **0** | **1.0000** |  | **0** | **0** | **1.0000** |  | **3** | **0** | **0.1143** |
| **52** | **2** | **2** | **0.0000** | **1.0000** |  | **0** | **0** | **1.0000** |  | **0** | **0** | **1.0000** |  | **1** | **0** | **0.5000** |
| **55** | **4** | **5** | **0.1132** | **0.7365** |  | **0** | **0** | **1.0000** |  | **0** | **0** | **1.0000** |  | **1** | **2** | **0.3857** |
| **56** | **2** | **2** | **0.0000** | **1.0000** |  | **0** | **0** | **1.0000** |  | **0** | **1** | **0.5000** |  | **0** | **0** | **1.0000** |
| **57** | **9** | **6** | **0.6192** | **0.4313** |  | **0** | **0** | **1.0000** |  | **0** | **1** | **0.5000** |  | **0** | **4** | **0.0519** |
| **58** | **2** | **2** | **0.0000** | **1.0000** |  | **0** | **1** | **0.5000** |  | **0** | **0** | **1.0000** |  | **2** | **2** | **0.3974** |
| **60** | **13** | **16** | **0.3301** | **0.5656** |  | **0** | **0** | **1.0000** |  | **0** | **0** | **1.0000** |  | **3** | **0** | **0.1143** |
| **61** | **3** | **2** | **0.2021** | **0.6530** |  | **0** | **1** | **0.5000** |  | **0** | **0** | **1.0000** |  | **3** | **0** | **0.1143** |
| **62** | **15** | **18** | **0.2927** | **0.5885** |  | **0** | **1** | **0.5000** |  | **0** | **0** | **1.0000** |  | **4** | **0** | **0.0519** |
| **64** | **1** | **4** | **1.8188** | **0.1775** |  | **0** | **0** | **1.0000** |  | **0** | **0** | **1.0000** |  | **0** | **0** | **1.0000** |
| **65** | **3** | **6** | **1.0189** | **0.3128** |  | **0** | **0** | **1.0000** |  | **0** | **0** | **1.0000** |  | **0** | **0** | **1.0000** |
| **70** | **1** | **1** | **0.0000** | **1.0000** |  | **0** | **0** | **1.0000** |  | **0** | **0** | **1.0000** |  | **0** | **0** | **1.0000** |
| **71** | **1** | **0** | **1.0021** | **0.3168** |  | **0** | **2** | **0.2429** |  | **0** | **0** | **1.0000** |  | **0** | **1** | **0.5000** |
| **72** | **0** | **0** | **0.1000** | **0.7518** |  | **0** | **1** | **0.5000** |  | **0** | **0** | **1.0000** |  | **0** | **0** | **1.0000** |
| **73** | **0** | **0** | **0.1000** | **0.7518** |  | **0** | **1** | **0.5000** |  | **0** | **0** | **1.0000** |  | **0** | **0** | **1.0000** |
| **81** | **0** | **0** | **0.1000** | **0.7518** |  | **0** | **2** | **0.2429** |  | **0** | **0** | **1.0000** |  | **0** | **0** | **1.0000** |

Supplemental Table 11. HLA-C Locus Distributions by Race/Ethnicity of Patients with ASD Indicates that HLA-Cw7 is Found Expressed More Frequently than from the Expected Distributions in Caucasian Subjects. One hundred forty three subjects with ASD were studied, providing 286 HLA alleles. The expected ASD number for the HLA-C locus allele is calculated by adjusting the known race/ethnic frequency of the specific HLA allele with the frequency found in the ASD study population. This was then further divided into specific race/ethic groups, providing 242 alleles in Caucasians, 18 alleles in African Americans, 8 alleles in Hispanics, and 18 alleles in Asians with ASD. The observed is the actual number of occurrences of the specific allele. *Chi square analysis was used to determine whether any allele was found in higher of lower proportions from the expected for Caucasians, and **Fisher Exact test was used for the remainder, with significance set at p<0.001. HLA-Cw7 was found to be statistically significantly in more Caucasian subjects with ASD.

|  | **Caucasian alleles=242** | | | |  | **African American alleles=18** | | |  | **Hispanic alleles=8** | | |  | **Asian alleles=18** | | |
| --- | --- | --- | --- | --- | --- | --- | --- | --- | --- | --- | --- | --- | --- | --- | --- | --- |
| **C**  **Locus** | **ASD Exp** | **ASD Obs** | **Chi Sq** | ***p =** |  | **ASD Exp** | **ASD Obs** | ****p =** |  | **ASD Exp** | **ASD Obs** | ****p =** |  | **ASD Exp** | **ASD Obs** | ****p =** |
| **1** | **7** | **6** | **0.0790** | **0.7786** |  | **0** | **2** | **0.2429** |  | **0** | **1** | **0.5000** |  | **5** | **1** | **0.0792** |
| **2** | **8** | **9** | **0.0610** | **0.8050** |  | **1** | **1** | **0.5143** |  | **0** | **0** | **1.0000** |  | **0** | **0** | **1.0000** |
| **4** | **27** | **27** | **0.0000** | **1.0000** |  | **1** | **2** | **0.3857** |  | **0** | **1** | **0.5000** |  | **3** | **2** | **0.3312** |
| **5** | **23** | **13** | **3.0010** | **0.0832** |  | **0** | **2** | **0.2429** |  | **0** | **3** | **0.1000** |  | **0** | **0** | **1.0000** |
| **6** | **21** | **18** | **0.2510** | **0.6164** |  | **1** | **0** | **0.5000** |  | **0** | **2** | **0.2333** |  | **1** | **5** | **0.0792** |
| **7** | **35** | **84** | **26.7546** | **0.0000002** |  | **1** | **2** | **0.3857** |  | **0** | **0** | **1.0000** |  | **9** | **5** | **0.1097** |
| **8** | **9** | **10** | **0.0548** | **0.8149** |  | **0** | **0** | **1.0000** |  | **0** | **1** | **0.5000** |  | **4** | **0** | **0.0519** |
| **9** | **17** | **17** | **0.0000** | **1.0000** |  | **0** | **1** | **0.5000** |  | **0** | **0** | **1.0000** |  | **2** | **1** | **0.3857** |
| **10** | **20** | **23** | **0.2297** | **0.6317** |  | **0** | **3** | **0.1143** |  | **0** | **0** | **1.0000** |  | **7** | **2** | **0.0517** |
| **12** | **14** | **14** | **0.0000** | **1.0000** |  | **0** | **1** | **0.5000** |  | **0** | **0** | **1.0000** |  | **2** | **2** | **0.3974** |
| **14** | **5** | **2** | **1.3046** | **0.2534** |  | **0** | **0** | **1.0000** |  | **0** | **0** | **1.0000** |  | **3** | **0** | **0.1143** |
| **15** | **6** | **3** | **1.0189** | **0.3128** |  | **0** | **2** | **0.2429** |  | **0** | **0** | **1.0000** |  | **1** | **0** | **0.5000** |
| **16** | **7** | **11** | **0.9232** | **0.3366** |  | **1** | **0** | **0.5000** |  | **0** | **0** | **1.0000** |  | **0** | **0** | **1.0000** |
| **17** | **4** | **3** | **0.1450** | **0.7034** |  | **0** | **0** | **1.0000** |  | **0** | **0** | **1.0000** |  | **0** | **0** | **1.0000** |
| **18** | **0** | **0** | **0.1000** | **0.7518** |  | **0** | **2** | **0.2429** |  | **0** | **0** | **1.0000** |  | **0** | **0** | **1.0000** |

Supplemental Table 12. HLA-DR Locus Distributions by Race/Ethnicity of Patients with ASD are not Statistically Significantly Different from the Expected Distributions. One hundred forty three subjects with ASD were studied, providing 286 HLA alleles. The expected ASD number for the HLA-DR locus allele is calculated by adjusting the known race/ethnic frequency of the specific HLA allele with the frequency found in the ASD study population. This was then further divided into specific race/ethic groups, providing 242 alleles in Caucasians, 18 alleles in African Americans, 8 alleles in Hispanics, and 18 alleles in Asians with ASD. The observed is the actual number of occurrences of the specific allele. *Chi square analysis was used to determine whether any allele was found in higher of lower proportions from the expected for Caucasians, and **Fisher Exact test was used for the remainder, with significance set at p<0.001. There were no statistically significant differences among the HLA-DR locus alleles.

|  | **Caucasian alleles=242** | | | |  | **African American alleles=18** | | |  | **Hispanic alleles=8** | | |  | **Asian alleles=18** | | |
| --- | --- | --- | --- | --- | --- | --- | --- | --- | --- | --- | --- | --- | --- | --- | --- | --- |
| **DR**  **Locus** | **ASD Exp** | **ASD Obs** | **Chi Sq** | ***p =** |  | **ASD Exp** | **ASD Obs** | ****p =** |  | **ASD Exp** | **ASD Obs** | ****p =** |  | **ASD Exp** | **ASD Obs** | ****p =** |
| **1** | **24** | **37** | **3.1700** | **0.0750** |  | **0** | **0** | **1.0000** |  | **0** | **0** | **1.0000** |  | **1** | **0** | **0.5000** |
| **4** | **41** | **31** | **1.6316** | **0.2015** |  | **0** | **3** | **0.1143** |  | **0** | **3** | **0.1143** |  | **6** | **2** | **0.0939** |
| **7** | **32** | **29** | **0.1688** | **0.6812** |  | **1** | **0** | **0.5000** |  | **0** | **3** | **0.1143** |  | **2** | **4** | **0.2404** |
| **8** | **8** | **10** | **0.2308** | **0.6309** |  | **0** | **0** | **1.0000** |  | **0** | **0** | **1.0000** |  | **3** | **0** | **0.1143** |
| **9** | **3** | **6** | **1.0189** | **0.3128** |  | **0** | **2** | **0.2429** |  | **0** | **0** | **1.0000** |  | **4** | **0** | **0.0519** |
| **10** | **2** | **2** | **0.0000** | **1.0000** |  | **0** | **2** | **0.2429** |  | **0** | **0** | **1.0000** |  | **1** | **1** | **0.5143** |
| **11** | **23** | **23** | **0.0000** | **1.0000** |  | **1** | **5** | **0.0792** |  | **0** | **0** | **1.0000** |  | **3** | **5** | **0.2310** |
| **12** | **4** | **4** | **0.0000** | **1.0000** |  | **0** | **0** | **1.0000** |  | **0** | **0** | **1.0000** |  | **4** | **1** | **0.1461** |
| **13** | **28** | **27** | **0.0205** | **0.8861** |  | **1** | **4** | **0.1461** |  | **0** | **0** | **1.0000** |  | **3** | **1** | **0.2494** |
| **14** | **6** | **8** | **0.2942** | **0.5875** |  | **0** | **1** | **0.5000** |  | **0** | **0** | **1.0000** |  | **3** | **0** | **0.1143** |
| **15** | **33** | **36** | **0.1521** | **0.6965** |  | **1** | **0** | **0.5000** |  | **0** | **2** | **0.2429** |  | **8** | **4** | **0.1070** |
| **16** | **3** | **5** | **0.5084** | **0.4758** |  | **0** | **1** | **0.5000** |  | **0** | **0** | **1.0000** |  | **0** | **0** | **1.0000** |
| **17** | **22** | **24** | **0.0961** | **0.7566** |  | **0** | **0** | **1.0000** |  | **0** | **0** | **1.0000** |  | **1** | **0** | **0.5000** |

Supplemental Table 13. HLA-DQ Locus Distributions by Race/Ethnicity of Patients with ASD are not Statistically Significantly Different from the Expected Distributions. One hundred forty three subjects with ASD were studied, providing 286 HLA alleles. The expected ASD number for the HLA-DQ locus allele is calculated by adjusting the known race/ethnic frequency of the specific HLA allele with the frequency found in the ASD study population. This was then further divided into specific race/ethic groups, providing 242 alleles in Caucasians, 18 alleles in African Americans, 8 alleles in Hispanics, and 18 alleles in Asians with ASD. The observed is the actual number of occurrences of the specific allele. *Chi square analysis was used to determine whether any allele was found in higher of lower proportions from the expected for Caucasians, and **Fisher Exact test was used for the remainder, with significance set at p<0.001. There were no statistically significant differences among the HLA-DQ locus alleles, although HLA-DQ5 and HLA-DQ6 approached statistical significance in Caucasians.

|  | **Caucasian alleles=242** | | | |  | **African American alleles=18** | | |  | **Hispanic alleles=8** | | |  | **Asian alleles=18** | | |
| --- | --- | --- | --- | --- | --- | --- | --- | --- | --- | --- | --- | --- | --- | --- | --- | --- |
| **DQ**  **Locus** | **ASD Exp** | **ASD Obs** | **Chi Sq** | ***p =** |  | **ASD Exp** | **ASD Obs** | ****p =** |  | **ASD Exp** | **ASD Obs** | ****p =** |  | **ASD Exp** | **ASD Obs** | ****p =** |
| **2** | **51** | **44** | **0.6418** | **0.4231** |  | **1** | **2** | **0.3857** |  | **0** | **1** | **0.5000** |  | **3** | **1** | **0.9860** |
| **4** | **6** | **9** | **0.6192** | **0.4313** |  | **0** | **0** | **1.0000** |  | **0** | **0** | **1.0000** |  | **3** | **0** | **2.9529** |
| **5** | **23** | **48** | **10.3161** | **0.0013** |  | **1** | **5** | **0.0792** |  | **0** | **0** | **1.0000** |  | **6** | **2** | **1.9442** |
| **6** | **36** | **63** | **9.2571** | **0.0023** |  | **1** | **4** | **0.1461** |  | **0** | **3** | **0.1143** |  | **5** | **4** | **0.1090** |
| **7** | **34** | **44** | **1.5284** | **0.2164** |  | **1** | **6** | **0.0400** |  | **0** | **1** | **0.5000** |  | **5** | **6** | **0.0895** |
| **8** | **14** | **17** | **0.3102** | **0.5776** |  | **0** | **1** | **0.5000** |  | **0** | **1** | **0.5000** |  | **1** | **2** | **0.3328** |
| **9** | **15** | **17** | **0.1338** | **0.7145** |  | **0** | **0** | **1.0000** |  | **0** | **2** | **0.2429** |  | **5** | **3** | **0.4895** |

| Supplementary Table 14: Discriminate Function Components | Component 1 | Component 2 | Total |
| --- | --- | --- | --- |
| Regression (PR) | -0.224608887 | -0.46443638 | -0.689045267 |
| Chronic Constipation (MR) | -0.678171191 | 0.02450488 | -0.653666311 |
| Headaches (PR) | -0.087129373 | -0.41138418 | -0.498513553 |
| Abdominal Pain (PR) | -0.24740178 | -0.21577999 | -0.46318177 |
| Anxiety (PR) | -0.055785538 | -0.01590746 | -0.071692998 |
| Chronic Persistent Infections (MR) | 0.313791827 | -0.38381075 | -0.070018923 |
| Immune Problems (PR) | -0.015329407 | 0.0446664 | 0.029336993 |
| Immune Disorder (MR) | 0.189947911 | -0.04859653 | 0.141351381 |
| Epilepsy (MR) | -0.003639808 | 0.19221935 | 0.188579542 |
| Allergies (PR) | 0.318787666 | -0.1288441 | 0.189943566 |
| Food Allergies/Intolerances (MR) | 0.3511555 | -0.02275702 | 0.32839848 |
| Sinusitis (PR) | -0.129901711 | 0.50134059 | 0.371438879 |
| ADHD (PR) | 0.202779472 | 0.3325216 | 0.535301072 |
