# Supplementary figures and images for: Inheritance of HLA-Cw7 Associated With Autism Spectrum Disorder (ASD)
Source: Front Psychiatry. 2019 Sep 11;10:612. doi: 10.3389/fpsyt.2019.00612 (PMC6749146; doi:10.3389/fpsyt.2019.00612)

PlotIndiv

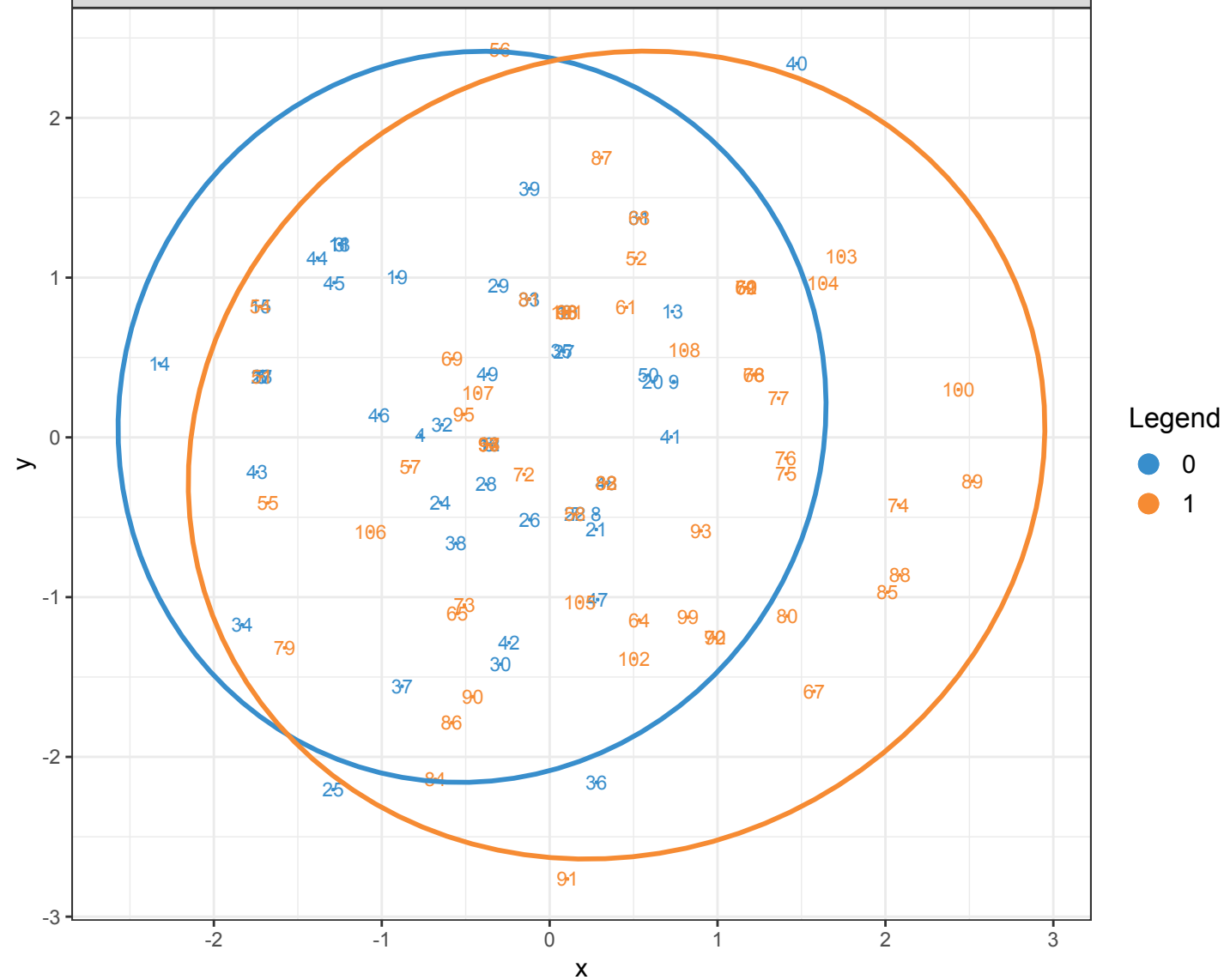

Supplement: Supplementary file 2 [file Image_1.pdf]

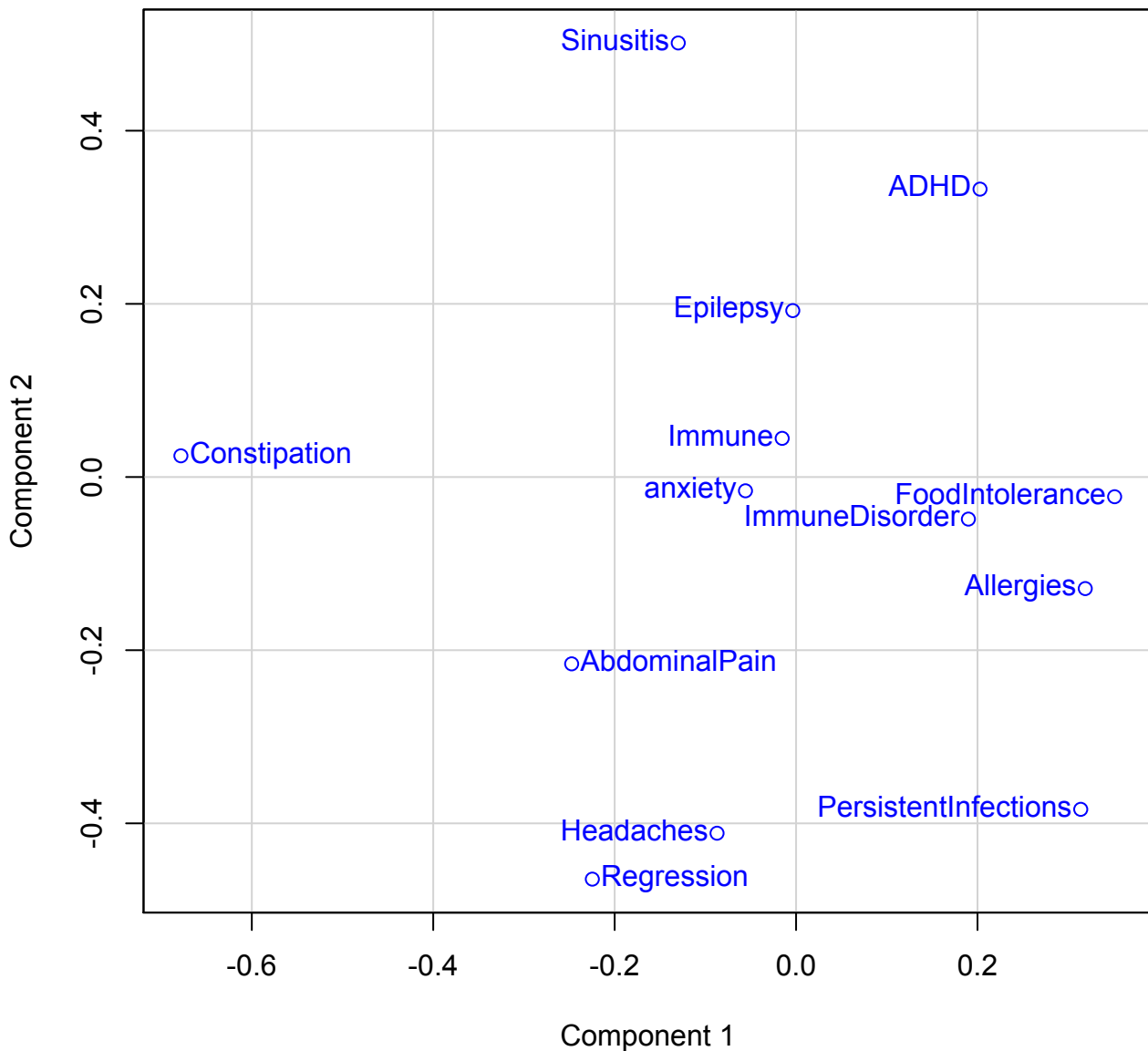

Supplement: Supplementary file 3 [file Image_2.pdf]
